# Long-term cardiac-specific mortality among 44,292 acute myeloid leukemia patients treated with chemotherapy: a population-based analysis

**DOI:** 10.7150/jca.36948

**Published:** 2019-10-15

**Authors:** Guangli Li, Zhijuan Zhou, Wencong Yang, Hao Yang, Xiuwu Fan, Yuelan Yin, Liyun Luo, Jinyou Zhang, Niujian Wu, Zibin Liang, Jianting Ke, Jian Chen

**Affiliations:** 1 Department of Cardiology, The Fifth Affiliated Hospital, Sun Yat-sen University, Zhuhai 519000, China.; 2 Center for Interventional Medicine, The Fifth Affiliated Hospital, Sun Yat-sen University, Zhuhai 519000, China.; 3 Department of Cardiology, The Seventh Affiliated Hospital, Sun Yat-sen University, Shenzhen 518017, Guangdong, China.; 4 Department of Thoracic Oncology, The Cancer Center of The Fifth Affiliated Hospital of Sun Yat-sen University, Zhuhai 519000, China.; 5 Department of Nephrology, The Fifth Affiliated Hospital, Sun Yat-sen University, Zhuhai 519000, China.; 6 Guangdong Provincial Key Laboratory of Biomedical Imaging, The Fifth Affiliated Hospital, Sun Yat-sen University, Zhuhai 519000, China.

**Keywords:** acute myeloid leukemia, chemotherapy, cardiac-specific death, epidemiology and cohort study.

## Abstract

**Background:** Acute myeloid leukemia (AML) is a common hematological malignancy treated with regimens containing anthracycline, an agent with cardiotoxicity. However, the cardiac-specific mortality in AML patients receiving chemotherapy remains unknown.

**Methods:** In this population-based study, patients diagnosed with AML between 1973 and 2015 were identified in the Surveillance, Epidemiology, and End Results database. Cumulative mortality by cause of death was calculated. To quantify the excessive cardiac-specific death compared with the general population, standardized mortality ratios (SMRs) were calculated. Multivariate Cox regression analyses were performed to identify risk factors associated with cardiac-specific death and AML-specific death.

**Results:** A total of 64,679 AML patients were identified between 1973 and 2015; 68.48% of patients (44,292) received chemotherapy. Among all possible competing causes of death, AML was associated with the highest cumulative mortality. The AML patients who received chemotherapy showed excessive cardiac-specific mortality compared with the general population, with an SMR of 6.35 (95% CI: 5.89-6.82). Age, year of diagnosis, sex, and marital status were independently associated with patient prognosis.

**Conclusion:** Cardiac-specific mortality in AML patients receiving chemotherapy is higher than that in the general population.

## Introduction

Acute myeloid leukemia (AML) is a malignancy originating from abnormally differentiated blasts of myeloid lineage 1. It has been estimated that 61,780 new leukemia cases and 22,840 leukemia-related deaths will be observed in the U.S.A. in 2019 2. Previous reports showed that AML usually occurs in elderly adults, of whom the median age is 67 years, and approximately 30% of AML patients are over 75 years old 3. Over the past decades, development in hematopoietic stem cell transplantation, supportive care and salvage chemotherapy has led to the prolongation of patient survival 4, 5. The first-line induction chemotherapy regimen for AML, except for acute promyelocytic leukemia (APL), has been the daunorubicin and cytarabine (DA) regimen for more than three decades, and this anthracycline-containing regimen is believed to exert cardiac toxicity 6. Previous studies on the adverse effect of anthracycline on heart physiology demonstrated that it may cause arrhythmia and congestive heart failure in patients with breast cancer and Hodgkin lymphoma 7-11. Furthermore, a recent publication demonstrated that it may lead to left ventricular function impairment in children survivors of AML 12. The treatment of APL has developed dramatically over the past three decades due to the elucidation of the PML/RAR fusion gene, from anthracycline monotherapy to arsenic-based therapy to a combinatorial therapy of arsenic and all-trans retinoic acid 13. However, arsenic agents have been proven to impair cardiac electrophysiology and ultimately increase cardiac-specific mortality 14, 15. Therefore, it is thought that chemotherapy is of high risk for AML patients, a population with many senior citizens 16. Despite the fact that agents with heart toxicity are frequently used in AML, quantitative analyses of cardiac-specific mortality based on large sample sizes have not been reported. Thus, it is of great importance and urgency to clarify the cardiac-specific mortality in AML patients receiving chemotherapy.

Previous reports in this field mainly focused on the molecular interactions of heart toxicity, potential biomarkers, and preventive agents or were simply case reports 17-23. It is also important to clarify the epidemiology of chemotherapy-related cardiac-specific death in AML patients to find some clues for basic research. The current study utilized data from the population-based Surveillance, Epidemiology, and End Results (SEER) database to report cardiac-specific mortality by age and to identify risk factors.

## Materials and methods

### Case inclusion

We accessed the SEER database, which is maintained and updated by the National Cancer Institute, to enroll AML cases. For all incident cases in the coverage areas, the SEER registries collect clinicopathological and demographic information, such as sex, race, age, socioeconomic status (SES), survival time, and vital status 24. The 18 registries of the SEER database cover approximately 28% of the total population, with nine registries covering 9% of the total population 25.

Data collection was performed by National Cancer Institute SEER*Stat software (seer.cancer.gov/seerstat) version 8.3.2. Cases of AML were histologically identified by the following ICD-O-3 codes: 9840/3, 9860/3, 9861/3, NOS, 9865/3, 9866/3, 9867/3, 9869/3, 9870/3, 9871/3, 9872/3, 9873/3, 9874/3, 9891/3, 9895/3, 9896/3, 9897/3, 9898/3, 9910/3, 9911/3, 9920/3 and 9931/3. We identified patients with a first malignancy diagnosis of AML between 1 January 1973 and 31 December 2015. We excluded patients who were diagnosed only by death certificate or autopsy report and patients whose age, race, SES status or survival time were unknown. The county-level SES was used as a surrogate of personal SES for each case and was defined as the percentage of residents living below the national poverty threshold in the 2000 U.S. Census 26-28. Additionally, three levels of SES were defined by thresholds recommended by the National Cancer Institute: <10% for low-poverty areas, 10%-19.99% for medium-poverty areas, and >20% for high-poverty areas 29.

### Statistical analyses

The baseline characteristics of the included cases are summarized in Table 1, with frequencies for categorical variables and means and corresponding standard deviations for continuous variables; their differences were tested by chi-square analyses and unpaired t tests, respectively. Cumulative mortality was computed for each cause of death and grouped by age. As the mortality of cardiac-specific diseases increases significantly with age in the general population, we calculated the standardized mortality ratios (SMRs) with respect to the mortality data for each age category of the general population in the U.S.A. to address this problem 30. We divided the observed number of cardiac-specific deaths by the expected number in the same age category, which was obtained by multiplying the cumulative person-time in AML patients in an age group with the age-specific cardiac-specific mortality rates for the general population in the same age group. All age-specific SMRs were computed for patients older than 50 years receiving chemotherapy. The 95% confidential intervals and p-values were also calculated 31, 32. Multivariate Cox regression analyses were performed to identify the risk factors for both cardiac-specific and AML-specific death. All other analyses were performed with Stata software, version 14.0 (StataCorp. 2015. Stata Statistical Software: Release 14. College Station, TX: StataCorp LP.). A two-sided p < 0.05 was considered statistically significant.

## Results

### Baseline characteristics

Of the 64,679 patients diagnosed with acute myeloid leukemia (AML) between 1973 and 2015, 68.48% (44,292) received chemotherapy, and 31.52% of patients (20,387) did not receive chemotherapy (Table 1). Patients who received chemotherapy were generally younger at diagnosis, with an average age of diagnosis of 54 years, compared with those in the nonchemotherapy group (average age: 73 years old). Additionally, about 86% of patients in the nonchemotherapy group were older than 60 years compared with 47.48% in the chemotherapy group. The majority of patients were white, and the sex, socioeconomic status (SES) and marital status distributions were relatively homogeneous in both groups. In terms of the calendar period of diagnosis, patients diagnosed in the last decade were more likely to receive chemotherapy than those diagnosed prior (46.22% vs. 39.92%). Generally, patients who received chemotherapy had a higher probability of survival (26.74% vs. 4.85%) and a lower probability of dying from AML (62.39% vs. 78.36%) or heart disease (1.71% vs. 4.19%).

### Cumulative mortality

**Figure 1:**
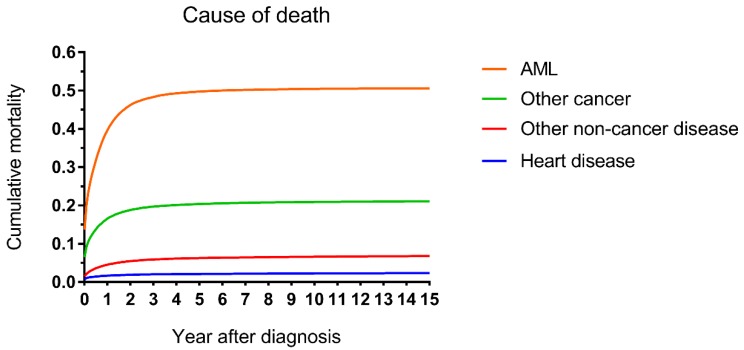
Cumulative mortality by causes of death in patients with AML receiving chemotherapy from 1973 to 2015 in SEER 18 registries.

**Figure 2:**
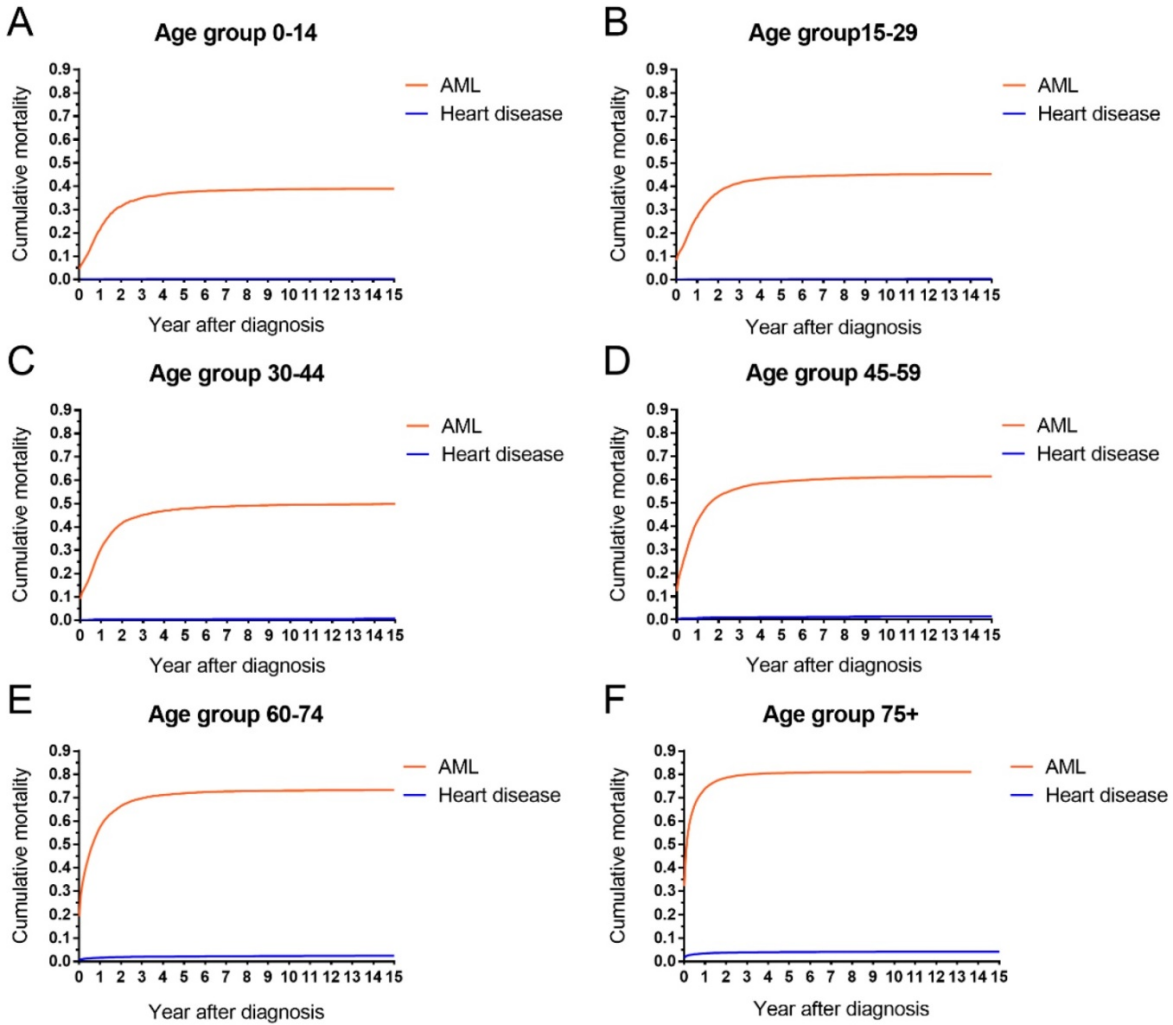
AML-specific and cardiac-specific mortality by age in patients with AML receiving chemotherapy from 1973 to 2015 in SEER 18 registries.

**Figure 3:**
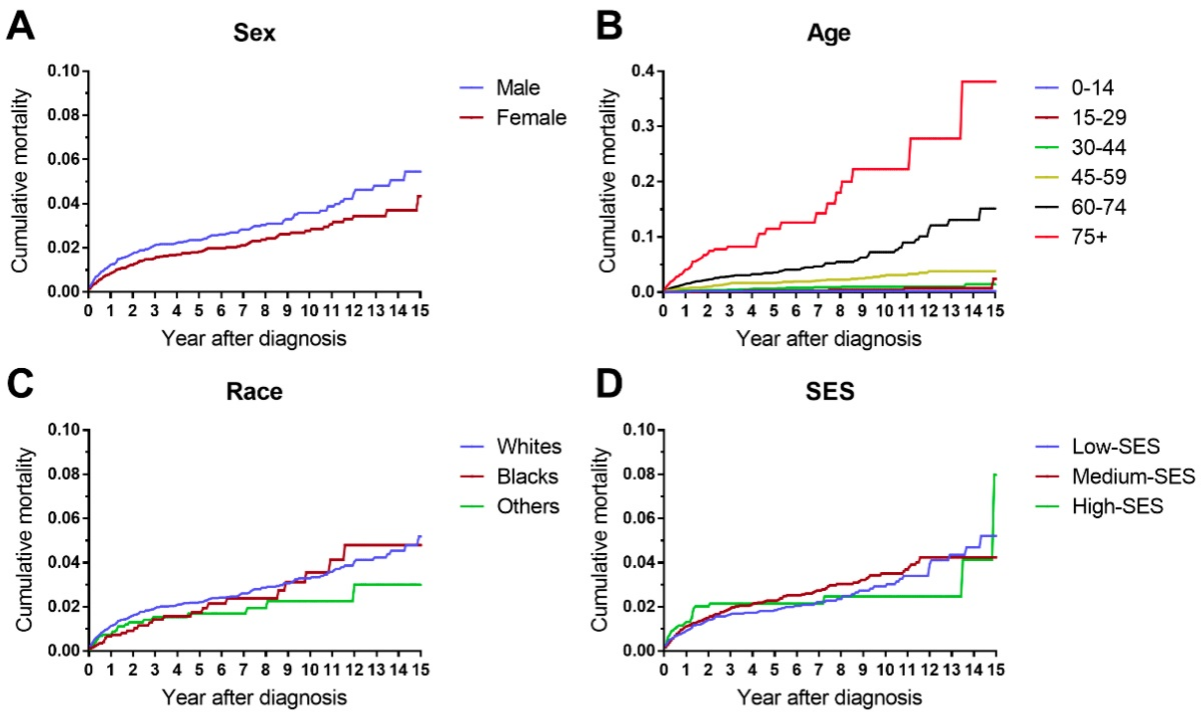
Differences in cardiac-specific mortality by sex, age, race and socioeconomic status in patients with AML receiving chemotherapy from 1973 to 2015 in SEER 18 registries.

The cumulative mortalities of AML patients receiving chemotherapy are illustrated by causes of death in Figure 1. AML was the major cause of death with the highest mortality, followed by other cancers, other noncancer diseases and heart disease. In addition, the heart disease mortality marginally increased over the 15-year period after diagnosis. In all age groups, the highest mortality rates were found in patients whose deaths were attributed to AML (Figure 2). Intuitively, the cumulative mortality of cardiac-specific disease increased with age at diagnosis and follow-up time. In terms of cardiac-specific cumulative mortality for AML patients who received chemotherapy, male patients showed higher cumulative mortality than female patients (Figure 3A). In terms of age, higher age was intuitively associated with higher cumulative mortality, with dramatically higher mortality in patients aged 60-74 and 75+ years (Figure 3B). White patients and Black patients showed similar mortality compared with the lower mortality in patients of other races (Figure 3C). Patients from medium-poverty regions showed higher mortality than those from low-poverty regions (Figure 3D).

### Standardized mortality ratios

The standardized cardiac-specific mortality of AML patients was calculated with regard to the general population (Table 2). For cardiac-specific death among AML patients receiving chemotherapy, the SMRs were dramatically high in AML patients for most age groups, with an overall cardiac-specific mortality of 6.35 (95% CI: 5.89-6.82). After stratification by age, the SMR decreased with age, from 11.03 (95% CI: 7.91-14.15) in patients aged 50-54 to 1.25 (95% CI: 0.95-1.56) in patients aged 80-84. Counterintuitively, patients who received chemotherapy showed a lower SMR of 2.26 (95% CI: 2.10-2.42) compared with counterparts who did not receive chemotherapy (SMR of 5.53, 95% CI: 5.27-5.79). In terms of all-cause mortality, the overall SMR was 95.80 (95% CI: 94.74-96.86) in the AML population. Similar decreasing trends of SMRs with age were also observed, from 144.13 in patients aged 50-54 to 15.08 in patients aged 80-84. Patients who received chemotherapy showed a lower SMR of 96.62 (95% CI: 95.57-97.67) compared with their nonchemotherapy counterparts with (SMR of 125.49, 95% CI: 124.24-126.73).

### Cause-specific hazard ratios

The correlation between clinicopathological characteristics and either cardiac-specific or AML-specific causes of death for patients who received chemotherapy is demonstrated in Table 3. Increased age was associated with higher risk for cardiac-specific death, with hazard ratios (HRs) of 1.34 (95% CI: 0.69-2.60), 3.33 (95% CI: 1.85-6.00), 8.91 (95% CI: 5.13-15.48), 26.45 (95% CI: 15.29-45.75), and 62.63 (95% CI: 35.83-109.48) for patients aged 15-29, 30-44, 45-59, 60-74, and 75+, respectively, compared with patients aged 0-14. However, the impact of age on AML-specific mortality was weaker than on cardiac-specific mortality, with hazard ratios of 1.22 (95% CI: 1.13-1.32), 1.53 (95% CI: 1.42-1.64), 2.35 (95% CI: 2.19-2.52), 3.83 (95% CI: 3.58-4.10), and 6.43 (95% CI: 6.00-6.90) for patients aged 15-29, 30-44, 45-59, 60-74, and 75+, respectively, compared with patients aged 0-14. In terms of the year of diagnosis, intuitively, patients diagnosed in later decades are associated with lower hazard ratios for both cardiac-specific and AML-specific mortality. For cardiac-specific mortality, hazard ratios were 0.71 (95% CI: 0.55-0.92), 0.46 (95% CI: 0.36-0.58), and 0.42 (95% CI: 0.33-0.53) for patients diagnosed in 1986-1995, 1996-2005, and 2006-2015, respectively, with patients diagnosed in 1973-1985 as a reference. For AML-specific mortality, hazard ratios were 0.75 (95% CI: 0.72-0.79), 0.58 (95% CI: 0.56-0.60), and 0.42 (95% CI: 0.40-0.44) for patients diagnosed in 1986-1995, 1996-2005, and 2006-2015, respectively, with patients diagnosed in 1973-1985 as a reference. No difference in risk of cardiac-specific mortality was observed among races. For AML-specific mortality, Black patients showed a marginally higher hazard ratio of 1.09 (95% CI: 1.04-1.14) compared with the hazard ratio of 0.55 (95% CI: 0.37-0.80) for patients of unknown race. In terms of sex, females showed lower hazard ratios in both cardiac-specific and AML-specific mortality (0.67 (95% CI: 0.57-0.79) and 0.89 (95% CI: 0.87-0.91), respectively), compared with males. For SES, a higher risk of AML-specific death was found in the medium- and high-poverty groups, with HRs of 1.04 (95% CI: 1.01-1.07) and 1.17 (95% CI: 1.11-1.24), respectively. In terms of marital status, unmarried patients were associated with a higher risk of cardiac-specific death, with an HR of 1.13 (95% CI: 1.10-1.16).

## Discussion

The current study is the first to demonstrate the mortality of AML patients by cause of death and extrapolate the cardiac-specific mortality. We found that cardiac-specific mortality accounts for approximately 2.50% of AML patient deaths, with a standardized mortality ratio of 6.35, implying a higher risk of death from heart disease compared with the general population.

Here, higher cardiac-specific mortality in AML patients who received chemotherapy was confirmed with an SMR of 6.35 compared with the general population. AML patients show increased cardiac-specific mortality as a result of both tumor cell infiltration in heart tissue and the administration of cardiotoxic chemotherapy, especially anthracycline-containing induction therapy 33-35. The cardiotoxicity of anthracycline increases in a cumulative dose-related manner, and therefore, a maximally tolerated dose was set as a cumulative dose of 550 mg/m2 to achieve a balance between tumor control and cardiac function impairment 36. In a study of induction chemotherapy, chemotherapy and cytokines released from the lysis of tumor cells may have instigated congestive heart failure in 18 out of 217 patients (8.29%); 5 of these patients recovered, whereas the remaining 13 patients did not (median survival of 71 days) 37. Basic research studies have shown that anthracycline can also cause myocyte necrosis 38, 39. These diverse detrimental effects are associated with polymorphisms of anthracycline metabolism enzymes, which makes tailored safety dosing possible 12, 21, 40, 41. Although the mechanism of cardiotoxicity in AML has not been clarified, studies in breast cancer patients whose chemotherapy regimens contain anthracycline demonstrated that the impairment of heart cells is mediated by stress protective signaling and reactive oxygen 42, 43. Notably, cardiac damage does not necessarily lead to cardiac-specific death, and thus, the mortality observed here may underestimate the cardiac damage. Therefore, future studies using ultrasound, MRI or PET as surveillance methods are of importance to provide a more precise landscape of heart damage. To study the excessive cardiac mortality that might be induced by chemotherapy, we calculated the SMRs, taking into account the increasing mortality from heart diseases with age in the general population. In the current study, we found a significantly higher SMR in patients who received chemotherapy and in each age category. Counterintuitively, patients who received chemotherapy showed a lower SMR compared with patients without chemotherapy. This result must be interpreted with caution, as there are differences in baseline characteristics, with generally higher ages and higher unmarried rates, in patients without chemotherapy. It has been reported that patients who receive chemotherapy are generally younger and have fewer comorbidities and better health 44-46.

In multivariate Cox regression analyses, the risks of death from heart diseases and AML increased with age, which seemingly contradicts the decreasing SMRs. This can be partially explained by the fact that the SMR was calculated by dividing the observed number of deaths in the AML cohort by the expected number of deaths in the general population who also show increasing mortality with increasing age. In terms of sex, females showed a reduced risk of cardiac-specific death compared with males, implying intrinsic biological differences in cardiac-specific disease mortality 16, 47. In terms of marital status, unmarried patients and patients with unknown marital status showed a higher risk of death compared with married patients, in line with a previous publication 48. This survival disparity may be associated with the impact of better social support on treatment adherence; living in a close and cohesive family increases the probability of adherence 1.7-fold, whereas living in an unstable family increases the risk of nonadherence 1.5-fold 49. In terms of SES, residing in high-poverty regions was marginally associated with a higher risk of cardiac-specific death, which implies poor healthcare accessibility in poor regions 50, 51. In AML-specific mortality, elderly patients and patients diagnosed in early decades had a higher risk of death. The higher risk of death in elderly patients may be attributable to treatment-related mortality and resistance to chemotherapy 52, 53. Elder patients are less able to tolerate intensive chemotherapy because of poor performance status, more comorbidities and poor immunity towards posttreatment infection. Additionally, older individuals show a high incidence of unfavorable genetic mutations and treatment-resistant phenotypes 54-56. Importantly, Black patients have a higher risk of death due to treatment differences and unmeasured genetic differences associated with excessive mortality during induction 57, 58. A lower risk of AML-specific death in females may imply a protective role of estrogen 59, 60. Inferior survival in patients from medium-poverty and high-poverty regions implies the impact of SES on survival via the limitation of access to healthcare resources, such as adequate treatment and surveillance 26, 61, 62.

The current study has strengths as well as limitations. First, the SEER database does not provide detailed information about the chemotherapy regimen, chemotherapy duration or application of hematopoietic stem cell transplantation, which have also been proven to be harmful to heart function 63, 64. Moreover, the SEER database does not provide detailed information, such as comorbidities, types of heart disease, and menopause status of females, which would be helpful for further analysis. Second, due to the long-term follow-up of these patients, the hazard ratios here imply the average risk of a specific group. Third, the results in the current study may be biased by confounding factors owing to its retrospective nature. As all cases came from the 18 registries of the SEER database, the epidemiological trend here can only reflect the pattern in the selected areas, and great heterogeneity exists in different patient populations in terms of AML biology (different mutation patterns), healthcare services, and chemotherapy dosages based on physicians' preference. Therefore, caution should be used when interpreting the current results to predict the epidemiology of AML in other regions. Fourth, the calculation of SMRs did not adjust for other confounders, as we did in the multivariate Cox regressions, which may confound these results. Fifth, the standard mortality data of the general population from CDC WONDER are provided in a combined manner, and the exclusion of persons diagnosed with AML was not possible. However, due to the low incidence of AML (nearly 4 per 100,000) in the general population, our results may not be substantially biased. Last, the representativeness of the SEER database for the general population in the U.S.A. is moderately questioned, as the population in SEER registries are shown to have a lower SES and more diverse composition of ethnic minorities 65.

In summary, the current study distinguishes itself by the large sample size and long follow-up time, which make up for the limitations of not having a randomized clinical trial on this topic. Studying the long-term cardiac-specific mortality in AML patients may help to better tailor clinical surveillance protocols and ultimately improve patient wellbeing.

## Figures and Tables

**Table 1:** Basic characteristics of all AML cases by chemotherapy status between 1973 and 2015. Data are number of patients in each category, with percentage in parentheses.

Category	All patients(n, %)	Chemotherapy(n, %)		P value
		yes	no	
	64679	44292(68.48)	20387(31.52)	
**Age**				
Mean ± SD	59.78 ± 21.96	53.56 ± 21.59	73.29 ± 15.84	< 0.01
0-14	3216 (4.97)	3004 (6.78)	212 (1.04)	< 0.01
15-29	4406 (6.81)	4037 (9.11)	369 (1.81)	
30-44	6727 (10.40)	6067 (13.70)	660 (3.24)	
45-59	11769 (18.20)	10156 (22.93)	1613 (7.91)	
60-74	19553 (30.23)	14000 (31.61)	5553 (27.24)	
75+	19008 (29.39)	7028 (15.87)	11980 (58.76)	
**Race**				
White	53766 (83.13)	36319 (82.00)	17447 (85.58)	< 0.01
Black	5342 (8.26)	3851 (8.69)	1491 (7.31)	
Other	5335 (8.25)	4007 (9.05)	1328 (6.51)	
Unknown	236 (0.36)	115 (0.26)	121 (0.59)	
**Sex**				
Male	35091 (54.25)	24395 (55.08)	10696 (52.46)	< 0.01
Female	29588 (45.75)	19897 (44.92)	9691 (47.54)	
**SES**				
Low	26922 (41.62)	18469 (41.70)	8453 (41.46)	=0.25
Medium	33693 (52.09)	23087 (52.12)	10606 (52.02)	
High	4064 (6.28)	2736 (6.18)	1328 (6.51)	
**Marriage**				
Married	34442 (53.25)	24724 (55.82)	9718 (47.67)	< 0.01
Unmarried	27659 (42.76)	18286 (41.29)	9373 (45.98)	
Unknown	2578 (3.99)	1282 (2.89)	1296 (6.36)	
**Year**				
1973-1985	7991 (12.35)	5138 (11.60)	2853 (13.99)	< 0.01
1986-1995	8138 (12.58)	5551 (12.53)	2587 (12.69)	
1996-2005	19939 (30.83)	13131 (29.65)	6808 (33.39)	
2006-2015	28611 (44.24)	20472 (46.22)	8139 (39.92)	
**Cause of death**				
Alive	12831 (19.84)	11842 (26.74)	989 (4.85)	< 0.01
AML	43609 (67.42)	27633 (62.39)	15976 (78.36)	
Cardiac	1614 (2.50)	759 (1.71)	855 (4.19)	
Other	6625 (10.24)	4058 (9.16)	2567 (12.59)	

Abbreviations: SD: standard deviation; SES: socioeconomic status; AML: acute myeloid leukemia.

**Table 2:** Standardized mortality rates for AML patients receiving chemotherapy by age and for all AML patients by chemotherapy status, relative to the USA general population. Data are standardized mortality rates, with 95% confidence interval in parentheses.

Groups	SMR cardiac-specific	P value	SMR overall	P value
Overall	6.35 (5.89-6.82)	< 0.0001	95.80 (94.74-96.86)	< 0.0001
Age				
50-54	11.03 (7.91-14.15)	< 0.0001	144.13 (138.33-149.92)	< 0.0001
55-59	9.98 (7.74-12.23)	< 0.0001	107.54 (103.65-111.43)	< 0.0001
60-64	6.13 (4.82-7.44)	< 0.0001	77.76 (75.24-80.28)	< 0.0001
65-69	5.11 (4.16-6.05)	< 0.0001	55.80 (54.09-57.50)	< 0.0001
70-74	3.26 (2.66-3.86)	< 0.0001	37.61 (36.47-38.75)	< 0.0001
75-79	1.86 (1.48-2.24)	< 0.0001	24.13 (23.32-24.94)	< 0.0001
80-84	1.25 (0.95-1.56)	= 0.077	15.08 (14.43-15.73)	< 0.0001
Treatment				
Chemotherapy	2.26 (2.10-2.42)	< 0.0001	96.62 (95.57-97.67)	< 0.0001
No chemotherapy	5.53 (5.27-5.79)	< 0.0001	125.49 (124.24-126.73)	< 0.0001

Abbreviations: SMR: standardized mortality rates; AML: acute myeloid leukemia.

**Table 3:** Multivariate Cox regression analyses for AML patients with chemotherapy by causes of death. Data are hazard ratios, with 95% confidence interval in parentheses.

Strata	N	Cause-specific hazards ratios			
		Cardiac	P-value	AML	P-value
**Age**					
0-14	3004	Reference		Reference	
15-29	4037	1.34 (0.69-2.60)	0.388	1.22 (1.13-1.32)	< 0.01
30-44	6067	3.33 (1.85-6.00)	< 0.01	1.53 (1.42-1.64)	< 0.01
45-59	10156	8.91 (5.13-15.48)	< 0.01	2.35 (2.19-2.52)	< 0.01
60-74	14000	26.45 (15.29-45.75)	< 0.01	3.83 (3.58-4.10)	< 0.01
75+	7028	62.63 (35.83-109.48)	< 0.01	6.43 (6.00-6.90)	< 0.01
**Year**					
1973-1985	5138	Reference		Reference	
1986-1995	5551	0.71 (0.55-0.92)	< 0.01	0.75 (0.72-0.79)	< 0.01
1996-2005	13131	0.46 (0.36-0.58)	< 0.01	0.58 (0.56-0.60)	< 0.01
2006-2015	20472	0.42 (0.33-0.53)	< 0.01	0.42 (0.40-0.44)	< 0.01
Race					
White	36319	Reference		Reference	
Black	3851	1.01 (0.75-1.36)	0.934	1.09 (1.04-1.14)	< 0.01
Other	4007	1.01 (0.77-1.33)	0.939	1.03 (0.98-1.08)	0.223
Unknown*	115	-	-	0.55 (0.37-0.80)	< 0.01
**Sex**					
Male	24395	Reference		Reference	
Female	19897	0.67 (0.57-0.79)	< 0.01	0.89 (0.87-0.91)	< 0.01
SES					
Low	18469	Reference		Reference	
Medium	23087	1.11 (0.95-1.31)	0.202	1.04 (1.01-1.07)	< 0.01
High	2736	1.42 (1.01-2.00)	0.044	1.17 (1.11-1.24)	< 0.01
**Marriage**					
Married	24724	Reference		Reference	
Unmarried	18286	1.56 (1.32-1.85)	< 0.01	1.13 (1.10-1.16)	< 0.01
Unknown	1282	1.42 (0.90-2.23)	0.13	0.97 (0.89-1.05)	0.443

Abbreviations: AML: acute myeloid leukemia; SES: socioeconomic status; *: hazard ratio is inapplicable, due to few observations in this category.
